# Cardioprotective response of remote ischemic preconditioning: Revealing possible role of cannabinoid type 2 receptor and AMPK-mediated autophagy in rats

**DOI:** 10.34172/jcvtr.025.33453

**Published:** 2025-12-17

**Authors:** Kuldeep Kumar, Harlokesh Narayan Yadav, Amteshwar Singh Jaggi, Leonid Maslov, Nirmal Singh

**Affiliations:** ^1^Department of Pharmaceutical Sciences and Drug Research, Punjabi University, Patiala, India; ^2^Department of Pharmacology, All India Institute of Medical Sciences (AIIMS), New Delhi, India; ^3^Cardiology Research Institute, Tomsk National Research Medical Center of the Russian Academy of Science, Tomsk, Russia

**Keywords:** Autophagy, Cardioprotection, CB_2_R, Langendorff, MIRI, RIPC

## Abstract

**Introduction:**

Remote ischemic preconditioning (RIPC) is a non-invasive, practically acceptable and applicable conditioning technique reported to confer cardioprotection in myocardial ischemia-reperfusion injury (MIRI). It is documented that cannabinoid B_2_ receptor (CB_2_R) plays crucial role in providing cardioprotection in various cardiovascular pathologies.

**Methods:**

MIRI was induced in the isolated hearts of Wistar rats by exposing them to global ischemia of 30 minutes followed by subsequent reperfusion with Kreb’s Henseleit (KH) buffer solution of 120 minutes after mounting on the Langendorff Power Lab apparatus. RIPC was applied by providing four alternate inter-spread cycles of 5 min non-lethal ischemia and 5 min reperfusion by tying the pressure cuff at the hind limb of the rats before isolation of hearts.

**Results:**

Ischemia-reperfusion injury (IRI) induced myocardial damage was manifested in terms of significant increase in infarct size, elevated levels of cardiac specific markers *i.e.* Lactate dehydrogenase-1 (LDH-1), Creatine kinase-MB (CK-MB), Cardiac troponin-I (C-tPn-I), altered hemodynamic parameters *i.e.* decreased heart rate (HR), coronary flow rate (CFR), left ventricular developed pressure (LVDP), rate pressure product (RPP),+dp/dt_max_, and -dp/dt_min_ and other biochemical markers including increased thiobarbituric acid reactive species (TBARS), decreased glutathione reductase (GSH), and catalase; markers of oxidative stress, increased tumor necrosis factor-α (TNF-α); inflammatory marker, transforming growth factor-β (TGF-β); fibrosis marker, Bax, and caspase-3; markers of apoptosis. RIPC significantly reduced the infarct size, LDH-1, and CK-MB release and C-tPn-I content. Moreover, RIPC significantly improved series of aforementioned hemodynamic as well as biochemical parameters. Pre-administration of AM-630 (selective CB_2_R antagonist; 0.5 and 1 mg/kg;*i.p.*) and BML-275 *i.e.* AMP activated protein kinase (AMPK) mediated autophagy inhibitor; 1.5 and 3 mg/kg;*i.p.*) substantially abrogated the cardioprotective response of RIPC.

**Conclusion:**

The current findings highlight the pivotal role of CB_2_R activation and AMPK activated autophagy in cardioprotective mechanism of RIPC against MIRI.

## Introduction

 Cardiovascular diseases (CVDs) remain the leading cause of global mortality, accounting for over 20 million deaths annually.^1-3^ Among them, acute myocardial infarction (AMI) is the most prevalent that results due to complete coronary artery obstruction.^4,5^ Reperfusion, though essential, can paradoxically exacerbate injury termed as myocardial ischemia-reperfusion injury (MIRI); eventually leads to cardiomyocyte damage.^6,7^ Besides, thrombolytic therapies, few non-pharmacological strategies including different conditioning techniques such as ischemic preconditioning (IPC), RIPC, ischemic postconditioning (IPostC) etc. have been developed to mitigate ischemia-reperfusion injury (IRI).

 Preclinical^8,9^ and clinical studies^10,11^ witnessed that IPC and IPostC offer cardioprotection in IRI. RIPC is a safer, non-invasive strategy comprising brief ischemia-reperfusion cycles in a remote organ to protect the heart from injury.^12^ In contrast, IPC must be applied before major ischemia and directly to the heart, making it invasive and less practical in unpredictable clinical scenarios.^13^

 Clinical studies evidenced the cardioprotective efficacy of RIPC in myocardial injury.^14-16^ Molecular signaling pathways including Janus kinase-signal transducer activated transaminase-3 (JAK-STAT3), receptor for advanced glycation end products-HMGB1 (RAGE-HMGB1), stromal cell derived factor-1α-cysteine-X-cysteine chemokine receptor type-4 (SDF-1α-CXCR4), *etc.*, are reported to play an intricate role in conferring cardioprotection through RIPC.^17-19^

 The endocannabinoid system, comprising cannabinoid receptor type-1 (CB_1_R) and CB_2_R^20^, is expressed in various organs including central nervous system (CNS), immune cells, and the heart (CB_2_).^20,21^ While CB_1_R is predominantly linked to adverse cardiac effects such as impaired contractility and lipid accumulation^22,23^, CB_2_R has gained attention for its cardioprotective potential in conditions like AMI and IRI. Although, CB_2_R conventionally studied in pain, obesity, and cognitive disorders^24^, the role of cannabinoid receptors in cardiovascular diseases is an emerging field, demands deeper investigation to clarify their complex effects on the heart. It is documented that CB_2_R activation attenuates inflammation, oxidative stress, and inhibit cardiomyocyte apoptosis.^25^ These cardioprotective effects occurs through modulation of intracellular signaling pathways including phospho-ionositol-3-kinase/Akt (PI3K/Akt), mitogen activated protein kinase (MAPK), and suppression of nuclear factor-κB (NF-κB) mediated inflammation.^26-28^ Additionally, endocannabinoids mediate the protective effects of IPC and RIPC through modulation of cannabinoid receptor dependent signaling pathways.^29,30^ However, the role of CB_2_R in cardioprotective effect mediated through RIPC is yet to be explored.

 Autophagy, a cellular process that degrades organelles and proteins^31^, occur in both normal and stress conditions like ischemia and hypoxia.^32^ Its regulation through various signaling pathways plays a key role in attenuating IRI.^33,34^ Notably, CB_2_R activation has been linked to autophagy-mediated cardioprotection in several pathological states.^35,36^ AMPK, a key energy sensor kinase regulates metabolism and autophagy.^37^ While AMPK-mediated autophagy is known to attenuate IRI in various organs^38-40^, its role in RIPC-induced cardioprotection during MIRI remains unexplored. Although, CB_2_R and AMPK-mediated autophagy have independently been implicated in cardioprotection, their simultaneous involvement in the context of RIPC-induced protection against MIRI has not been investigated. Therefore, the present study was undertaken to explore, for the first time, the combined role of CB_2_R and AMPK-autophagy in mediating the cardioprotective effects of RIPC using selective pharmacological inhibitors.

## Materials and Methods

###  Animals

 Wistar rats of either sex, weighing 150-200 g were utilized in the current study and were procured from Disease Free Small Animal House, Lala Lajpat Rai University of Veterinary and Animal Sciences (LUVAS), Hisar, Haryana. The experimental animals were maintained in the departmental animal facility under standard laboratory conditions, with free access to tap water and a regular diet (*Chao* feed, Ashirwad Industries, Chandigarh). A 12-hour light/dark cycle was maintained throughout the study. The protocols of experiments were approved by the Institutional Animal Ethics Committee (IAEC), (Reg. no. 107/Go/ReBi/S/99/CCSEA/2021-10), and animal care and handling were conducted in accordance with the guidelines issued by the Committee for Control and Supervision of Experiments on Animals (CCSEA), Ministry of Fisheries, Animal Husbandry and Dairying, Department of Animal Husbandry and Dairying, Govt. of India.

###  Drugs and Chemicals

 AM-630 and BML-275 were purchased from Cayman Chemicals and Aba Chem Scene Pvt. Ltd., USA respectively. Different chemicals including Dinitrophenylhydrazine (DNPH), Nicotinamide adenine dinucleotide (oxidized) (NAD^+^), Nicotinamide adenine dinucleotide (reduced) (NADH), lactate, Triphenyl tetrazolium chloride (TTC), 5,5-dithiobis-2-nitrobenzoic acid (DTNB), 2,4-thiobarbituric acid (TBA), Trichloroacetic acid (TCA), Hydrogen peroxide (H_2_O_2_) and Dimethyl sulfoxide (DMSO) were obtained from Loba Chemie Pvt Ltd., India. Bovine serum albumin (BSA) and Folin-Ciocalteu Reagent (FCR) were procured from Sisco Research Laboratories Pvt. Ltd., India. The chemicals employed in this study were of standard grade and were freshly prepared. CK-MB, C-tPn-I, TNF-α, TGF-β, Bax and Caspase-3 ELISA kits were purchased from Krishgen Biosystems, Mumbai, Maharashtra, India. AM-630 and BML-275 were dissolved in 10% DMSO and they were used as selective CB_2_R and AMPK mediated autophagy inhibitors respectively. The doses of AM-630 (0.5 and 1 mg/kg; *i.p.*) ^30,41^ and BML-275 (1.5 and 3 mg/kg; *i.p.*)^42^ were selected as per the previously published reports.

###  Experimental Model

 The rat was anesthetized using thiopental sodium at a dose of 50 mg/kg administered intraperitoneally. To deliver the RIPC stimulus, a blood pressure cuff was positioned around the upper portion of one hind limb, specifically at the inguinal region. Ischemia was induced by inflating the cuff to 150 mmHg for 5 minutes, followed by deflation to allow reperfusion for another 5 minutes. This cycle of limb ischemia and reperfusion was repeated four times consecutively to precondition the tissue against IRI. Pre-treated heparanized heart were excised and retrogradely perfused with KH buffer solution at a constant pressure of 70 mm Hg, maintained at pH 7.4, optimum temperature of 37ºC and bubbled with 95% O_2_ and 5% CO_2 _on the Langendorff apparatus. Ischemia-reperfusion induced myocardial injury was assessed by inserting a fluid filled latex balloon into the left ventricle to record the LVDP, + dp/dt_max,_ -dp/dt_min_ and HR using a pressure transducer (AD instruments, Australia). Flow rate at different time intervals was also assessed to measure the extent of injury to coronary vasculature.^43,44^

###  Measurement of Infarct Size

 Following reperfusion phase, the heart was carefully excised from the cannula and preserved overnight at 0°C. The frozen heart was then sliced into uniform sections, each approximately 2–3 mm thick. For infarct visualization, the tissue slices were incubated in 1% TTC solution prepared in 0.2 M Tris buffer (pH 7.4) at 37°C for 20 min. After staining, viable myocardial tissue turned red, while infarcted (non-viable) areas remained pale yellow, clearly reflects the extent of myocardial injury. Further, the extent of infarct was calculated by using the method as described previously through area method.^45,46^

###  Estimation of Biochemical Parameters 

####  Collection of Coronary Effluent

 Collection of coronary effluent was done to evaluate the enzymatic activities of LDH-1 and CK-MB. Samples were obtained during the stabilization phase (basal) and at various time points following reperfusion specifically at 5, 30, 60, and 120 minutes.

####  Collection of Supernatants from Heart Homogenate

 After completing the experimental procedures, the hearts were weighed and homogenized in phosphate-buffered saline (PBS, pH 7.4). The homogenates were then centrifuged at 3,500 rpm for 15 min. at 4°C. The resulting supernatants were collected and stored at -20°C for the biochemical estimation of various parameters, including C-tPn-I, total protein content, TBARS, GSH, Catalase, TNF-α, TGF-β, Bax and Caspase-3.

####  Estimation of LDH-I Enzyme Activity

 LDH-1 activity in the coronary effluent samples collected at various time frames (basel, and 5, 30, 60, and 120 minutes post-reperfusion) was estimated using 2,4-DNPH method, as described by King.^47^

####  Estimation of CK-MB Enzyme Activity

 The activity of CK-MB was measured in the samples of coronary effluent obtained at different time frames (basal, 5, and 30 min. after reperfusion) by utilizing standardized procedure of commercially available ELISA kit obtained from Krishgen Biosystems, Mumbai. The final absorbance to estimate the CK-MB enzyme activity was measured at 450 nm by using ELISA reader.^48^

####  Measurement of C-tPn-I Protein Content 

 C-tPn-I protein content was measured in the heart homogenate by using standardized methodology of commercially available ELISA kit procured from Krishgen Biosystems, Mumbai. The final absorbance to assess the C-tPn-I protein content was measured at 450 nm by using ELISA reader.^49^

####  Estimation of Heart Total Protein Content:

 The whole heart was utilized for estimation of total protein content by using the spectrophotometric technique (Shimadzu 1800, Japan) at 750 nm by the method of Lowry and coworkers.^50^

####  Measurement of Heart TBARS Levels:

 The malondialdehyde (MDA) levels were measured in the heart homogenates by using the spectrophotometric technique (Shimadzu 1800, Japan) at 532 nm to measure the oxidative stress.^51^

####  Estimation of Heart GSH Levels:

 The Ellman’s method was employed to estimate the GSH content in the cardiac tissue homogenate spectrophotometrically (Shimadzu 1800, Japan) at 412 nm to assess oxidative stress.^52^

####  Assessment of Heart Catalase Activity:

 The Catalase activity was quantified in the heart homogenates spectrophotometrically (Shimadzu 1800, Japan) at 570 using Sinha’s method (Sinha, 1972) with slight modification.^53^

####  Estimation of Heart TNF-α, TGF-β, Bax and Caspase-3 Content:

 TNF-α, TGF-β, Bax and Caspase-3 contents were estimated in the heart homogenate by using standardized method of commercially available ELISA kit purchased from Krishgen Biosystems, Mumbai. The final absorbance to measure the TNF-α, TGF-β, Bax and Caspase-3 content was determined at 450 nm by using ELISA reader.^54-56^

###  Experimental Protocol

 Presently, we employed a total of 66 animals and were divided into 11 different groups containing 6 rats in each group.

####  Group I- Normal Control

 Rat heart was mounted on Langendorff apparatus and was perfuse with KH solution for 170 min.

####  Group II- Vehicle Control

 Twenty minutes after intraperitoneal administration of 10% DMSO, the hearts were isolated, stabilized, and then perfused with KH solution for 150 min. using the Langendorff perfusion apparatus.

####  Group III- RIPC Sham Control 

 In anesthetized rats, a blood pressure cuff was positioned around the hind limb without inflation or deflation. After 40 min. the heart was excised and perfused using the Langendorff apparatus. Following a 20-min. stabilization period, the heart was subjected to 30 min. of global ischemia, followed by 120 min. of reperfusion.

####  Group IV- IRI Control

 The isolated hearts, after stabilization of 20 min., were subjected to global ischemia for 30 min. and subsequently reperfused with KH solution for 120 min.

####  Group V- RIPC Control

 A pressure cuff was tied on the hind limb of the animals and four alternating cycles of 5-min. ischemia and 5-min. reperfusion were given. Thereafter, the hearts were isolated and, following stabilization, subjected to 30 min. of global ischemia, followed by 120 min. of reperfusion using KH solution.

####  Group VI- AM-630 (LD)+RIPC

 Rats were heparinized followed by administration of CB_2_R selective antagonist *i.e.* AM-630 (0.5 mg/kg;*i.p.*). After 30 min., animals were anaesthetized by using thiopental sodium (50 mg/kg;*i.p.*) and rest of the procedure was same as described in group-V.

####  Group VII- AM-630 (HD)+RIPC

 After heparinization, rats were subjected to the administration of CB_2_R selective antagonist *i.e.* AM-630 (1 mg/kg;*i.p.*). After 30 min., animals were anaesthetized by using thiopental sodium (50 mg/kg;*i.p.*) and remaining procedure was similar as discussed in group-V.

####  Group VIII- BML-275 (LD)+RIPC

 Rats were heparinized followed by administration of selective AMPK inhibitor *i.e.* BML-275 (1.5 mg/kg;*i.p.*). After 30 min., animals were anaesthetized and then followed by same procedure as that of group-V.

####  Group IX- BML-275 (HD)+RIPC

 After heparinization, rats were subjected to the administration of selective AMPK inhibitor *i.e.* BML-275 (3 mg/kg;*i.p.*). After 30 min., animals were anaesthetized than same procedure was repeated as described in group-V,

####  Group X- AM-630 Per Se

 The administration of AM-630 (1 mg/kg;*i.p.*) was done, followed by the isolation of hearts. The isolated hearts, after stabilization, were perfused for a period of 150 min. with KH solution on the Langendorff perfusion apparatus.

####  Group XI- BML-275 Per Se

 Before isolation of hearts, administration of BML-275 (3 mg/kg;*i.p.*) was carried out. The isolated hearts, after stabilization, were subjected to perfusion for 150 min. with KH solution on the Langendorff perfusion apparatus.

###  Statistical Analysis

 Statistical analysis was performed using Graph Pad Prism software version 9.5.1 (733). Data are presented as mean ± standard deviation (SD), with six animals per group (n = 6). One-way and two-way analyses of variance (ANOVA) were used to assess statistical significance. One-way ANOVA was applied to infarct size (area method), TNF-α, TGF-β, Bax, Caspase-3, TBARS, GSH, and catalase levels. Two-way ANOVA was used for LDH-1, CK-MB, and hemodynamic parameters, including HR, LVDP, CFR, RPP, + dp/dt_max_, and -dp/dt_min_. For multiple group comparisons, Bonferroni *post hoc* test was applied following one-way ANOVA, while Tukey’s *post hoc* test was used after two-way ANOVA. A *P*-value of *P < 0.05 *was considered statistically significant.

## Results

###  Effects of various interventions on hemodynamic parameters

 Sustained prolonged ischemia of 30 min. followed by subsequent reperfusion of 120 min., significantly reduced the hemodynamic parameters including HR, LVDP, CFR, RPP, + dp/dt_max_, and -dp/dt_min_ in the IRI control group animals when compared to their basal values and the values of the normal and vehicle control group animals observed at different time intervals *i.e.* basal (stabilization period), 5, 30, 60, and 120 min., after reperfusion. In contrast, RIPC significantly improved all aforementioned hemodynamic parameters as compared to the IRI control group animals. On contrary, no significant improvements were observed in the RIPC sham control group animals as compared to the animals of IRI control group. However, the cardioprotective effects of RIPC were remarkably reversed by the pre-administration of AM-630 (a CB_2_R subtype selective antagonist; 0.5 and 1 mg/kg; *i.p.*) in dose dependent manner. Moreover, pre-treatment of BML-275 (an AMPK mediated autophagy inhibitor; 1.5 and 3 mg/kg; *i.p.*) also abolished the protective effects of RIPC in a significant manner (Table 1-5).

**Table 1:** Effects of Various Interventions on HR (beats/min.)

**Groups**	**Basal**	**5AR**	**30AR**	**60AR**	**120AR**
Normal Control	264.31 ± 6.83	271.55 ± 12.52	265.89 ± 7.70	268.37 ± 11.38	258.21 ± 10.46
Vehicle Control	272.5 ± 10.05	273.77 ± 11.43	267.22 ± 6.96	274.71 ± 10.61	261.09 15.33
RIPC Sham Control	263.88 ± 5.67	**105.65±7.85** ^ab*^	**126.99±6.72** ^ab*^	**148.51±7.00** ^ab*^	**131.87±7.26** ^ab*^
IRI Control	264.72 ± 7.30	**102.99±9.14** ^ab*^	**121.06±7.21** ^ab*^	**142.68±12.85** ^ab*^	**122.58±9.94** ^ab*^
RIPC Control	269.44 ± 12.10	**164.04±8.67** ^cd*^	**189.65±13.05** ^cd*^	**218.62±11.04** ^cd^	**198.55±12.91** ^cd*^
AM-630 (LD) + RIPC	260.48 ± 8.41	**126.79±4.37** ^abe*^	**145.18±9.43** ^abe*^	**168.42±7.76** ^abe*^	**151.46±12.56** ^ab*^
AM-630 (HD) + RIPC	259.83 ± 15.50	**109.05±4.13** ^abe*^	**134.53±10.47** ^9abe*^	**158.45±9.03** ^abe*^	**147.03±8.37** ^ab*^
BML-275 (LD) + RIPC	268.87 ± 9.08	**123.37±8.37** ^abe*^	**145.20±11.91** ^abe*^	**174.53±12.35** ^abe*^	**156.47±6.41** ^ab*^
BML-275 (HD) + RIPC	271.03 ± 5.62	**117.40±6.58** ^abe*^	**145.20±11.91** ^abe*^	**171.33±10.00** ^abe*^	**153.09±14.22** ^ab*^
AM-630 (HD) *per se*	269.53 ± 5.60	265.23 ± 8.34^cd^	268.92 ± 18.07^cd^	271.11 ± 9.48^cd^	262.96 ± 9.19^cd^
BML-275 (HD) *per se*	271.58 ± 14.48	263.10 ± 8.18^cd^	273.84 ± 11.39^cd^	268.90 ± 10.46^cd^	265.82 ± 6.94^cd^

Abbreviations: AR- After Reperfusion; IRI- Ischemia-Reperfusion Injury; RIPC- Remote Ischemic Preconditioning; LD- Low Dose (0.5 and 1.5 mg/kg;*i.p.*); HD- High Dose (1 and 3 mg/kg;*i.p.*)) Values are expressed as mean ± S.D. (n = 6). Basal denotes HR measured before global ischemia. 5AR, 30AR, 60AR, and 120AR denote HR measured 5, 30, 60, and 120 minutes after reperfusion following sustained global ischemia. * = *P* < 0.05 vs Basal;a = *P* < 0.05 vs Normal Control; b = *P* < 0.05 vs Vehicle Control; c = *P* < 0.05 vs IRI Control; d = *P* < 0.05 vs RIPC Sham Control; e = *P* < 0.05 vs RIPC Control. Bold numbers are statistically significant

**Table 2:** Effects of Various Interventions on LVDP (mmHg)

**Groups**	**Basal**	**5AR**	**30AR**	**60AR**	**120AR**
Normal Control	80.38 ± 3.54	82.57 ± 2.34	80.47 ± 4.00	82.02 ± 3.15	77.29 ± 3.37
Vehicle Control	81.56 ± 1.84	81.39 **±**2.55	82.49**±**2.67	81.49 **±**4.38	74.38**±**2.81
RIPC Sham Control	78.51 ± 2.16	**31.57±4.99** ^ab*^	**41.26±1.55** ^ab*^	**47.15±4.11** ^ab*^	**44.16±3.96** ^ab*^
IRI Control	80.16 ± 2.89	**27.64±2.17** ^ab*^	**40.24±3.97** ^ab*^	**44.00±2.92** ^ab*^	**40.67±4.93** ^ab*^
RIPC Control	81.40 ± 3.35	**49.11±4.47** ^cd*^	**62.84±1.54** ^cd*^	**68.54±4.02** ^cd*^	**61.25±2.55** ^cd*^
AM-630 (LD) + RIPC	79.72 ± 3.70	**34.02±4.56** ^abe*^	**48.52±2.54** ^abe*^	**51.80±5.09** ^abe*^	**46.23±3.78** ^ab*^
AM-630 (HD) + RIPC	81.19 ± 3.00	**30.46±2.00** ^abe*^	**45.47±2.74** ^abe*^	**48.95±4.01** ^abe*^	**43.19±1.64** ^abe*^
BML-275 (LD) + RIPC	80.47 ± 3.18	**35.52±3.77** ^abe*^	**47.18±3.37** ^abe*^	**49.90±3.59** ^abe*^	**47.76±2.95** ^ab*^
BML-275 (HD) + RIPC	79.58 ± 3.94	**30.95±4.22** ^abe*^	**44.81±2.21** ^abe*^	**47.89±5.42** ^abe*^	**45.99±3.53** ^abe*^
AM-630 (HD) *per se*	80.04 ± 3.92	79.50 **±**4.00^cd^	81.43 **±**3.82^cd^	81.76**±**2.43^cd^	75.31**±**2.40^cd^
BML-275 (HD) *per se*	79.58 ± 3.49	78.78 **±**4.34^cd^	80.62**±**4.52^cd^	80.02 **±**3.43^cd^	73.11**±**2.54^cd^

Abbreviations: AR- After Reperfusion; IRI- Ischemia-Reperfusion Injury; RIPC- Remote Ischemic Preconditioning; LD- Low Dose (0.5 and 1.5 mg/kg;*i.p.*); HD- High Dose (1 and 3 mg/kg;*i.p.*) Values are expressed as mean ± S.D. (n = 6). Basal denotes LVDP measured before global ischemia. 5AR, 30AR, 60AR, and 120AR denote LVDP measured 5, 30, 60, and 120 minutes after reperfusion following sustained global ischemia. * = *P* < 0.05 vs Basal;a = *P* < 0.05 vs Normal Control; b = *P* < 0.05 vs Vehicle Control; c = *P* < 0.05 vs IRI Control;d = *P* < 0.05 vs RIPC Sham Control; e = *P* < 0.05 vs RIPC Control. Bold numbers are statistically significant

**Table 3:** Effects of Various Interventions on CFR (mL/min.)

**Groups**	**Basal**	**5AR**	**30AR**	**60AR**	**120AR**
Normal Control	11.79 ± 1.00	12.29 ± 1.17	12.84 ± 0.39	12.92 ± 0.22	12.27 ± 0.34
Vehicle Control	11.97 ± 0.88	12.73**±**0.62	12.47**±**0.74	12.79**±**0.28	11.96**±**0.46
RIPC Sham Control	12.55 ± 0.58	**5.89±0.68** ^ab*^	**6.94±0.56** ^ab*^	**6.86±0.65** ^ab*^	**6.23±0.48** ^ab*^
IRI Control	12.31 ± 0.55	**4.96±0.58** ^ab*^	**6.13±0.68** ^ab*^	**6.73±0.52** ^ab*^	**6.42±0.26** ^ab*^
RIPC Control	11.99 ± 0.62	**10.15±0.35** ^cd*^	**10.61±0.37** ^cd*^	**11.46±0.73** ^cd^	**10.36±0.50** ^cd*^
AM-630 (LD) + RIPC	11.95 ± 1.09	**7.37±0.64** ^abe*^	**7.69±0.67** ^abe*^	**8.08±0.60** ^abe*^	**7.25±0.43** ^abe*^
AM-630 (HD) + RIPC	12.44 ± 0.72	**7.15±0.93** ^abe*^	**7.60±0.76** ^abe*^	** 7.85±0.35** ^abe*^	**6.78±0.67** ^abe*^
BML-275 (LD) + RIPC	11.94 ± 0.66	**7.52±0.60** ^abe*^	**7.85±0.60** ^abe*^	**8.24±1.06** ^abe*^	**7.18±0.47** ^abe*^
BML-275 (HD) + RIPC	11.87 ± 0.61	**7.24±0.57** ^abe*^	**7.76±0.72** ^abe*^	** 8.60±0.76** ^abe*^	**6.98±0.26** ^abe*^
AM-630 (HD) *per se*	12.57 ± 0.74	12.36**±**0.82^cd^	12.75**±**0.43^cd^	12.64**±**0.28^cd^	12.35**±**0.37^cd^
BML-275 (HD) *per se*	12.66 ± 0.83	12.79**±**0.36^cd^	12.88**±**0.55^cd^	12.76**±**0.37^cd^	12.15**±**0.50^cd^

Abbreviations: AR – After Reperfusion; IRI- Ischemia-Reperfusion Injury; RIPC- Remote Ischemic Preconditioning; LD- Low Dose (0.5 and 1.5 mg/kg;*i.p.*); HD- High Dose (1 and 3 mg/kg;*i.p.*) Values are expressed as mean ± S.D. (n = 6). Basal denotes CFR measured before global ischemia. 5AR, 30AR, 60AR, and 120AR denote CFR measured 5, 30, 60, and 120 minutes after reperfusion following prolonged global ischemia. * = *P* < 0.05 vs Basal;a = *P* < 0.05 vs Normal Control; b = *P* < 0.05 vs Vehicle Control; c = *P* < 0.05 vs IRI Control;d = *P* < 0.05 vs RIPC Sham Control; e = *P* < 0.05 vs RIPC Control. Bold numbers are statistically significant

**Table 4:** Effects of Various Interventions on RPP (beats.mmHg/min.)

**Groups**	**Basal**	**5AR**	**30AR**	**60AR**	**120AR**
Normal Control	21259.85 ± 1340.52	22394.74 ± 576.58	21419.37 ± 1547.15	22019.38 ± 1376.72	19957.29 ± 1246.35
Vehicle Control	22232.20 ± 1012.31	22272.90 **±**1020.91	22056.55**±**1200.01	22390.42**±**1585.04	19412.28**±**1230.12
RIPC Sham Control	20717.27 ± 720.35	**3357.02±728.69** ^ab*^	**5241.73±370.12** ^ab*^	**7014.47±801.73** ^ab*^	**5828.12±654.70** ^ab*^
IRI Control	21216.23 ± 917.65	**2858.09±413.20** ^ab*^	**4863.32±496.32** ^ab*^	**6252.70±422.57** ^ab*^	**4982.00±637.40** ^ab*^
RIPC Control	21935.30 ± 1379.69	**8082.02±1077.50** ^cd*^	**11918.53±896.34** ^cd^	**14954.07±642.10** ^cd*^	**12168.69±1022.92** ^cd^
AM-630 (LD) + RIPC	20763.66 ± 1182.07	**4320.73±629.55** ^abe*^	**7033.42±432.78** ^abe*^	**8716.13±897.03** ^abe*^	**6956.58±197.35** ^abe*^
AM-630 (HD) + RIPC	21113.57 ± 1727.89	**3317.22±197.41** ^abe*^	**6102.64±435.84** ^abe*^	**7752.07±750.01** ^abe*^	**6360.51±574.73** ^abe*^
BML-275 (LD) + RIPC	21636.35 ± 1156.29	**4398.50±639.08** ^abe*^	**7038.90±698.01** ^abe*^	**8709.95±901.03** ^abe*^	**7468.02±494.60** ^abe*^
BML-275 (HD) + RIPC	21549.07 ± 729.08	**3651.43±628.74** ^abe*^	**6503.40±576.39** ^abe*^	**8203.20± 1053.90** ^abe*^	**7022.35± 646.93** ^abe*^
AM-630 (HD) *per se*	21563.27 ± 857.41	21085.30**±**1064.32^cd^	21846.06**±**966.17^cd^	22160.08 **±**870.14^cd^	**19803.9±941.91** ^cd^
BML-275 (HD) *per se*	21598.01 ± 1322.49	20732.11 **±**1359.65^cd^	22093.58**±**1754.66^cd^	21531.21 **±**1455.86^cd^	19433.14**±**817.40^cd^

(Abbreviations: AR- After Reperfusion; IRI- Ischemia-Reperfusion Injury; RIPC- Remote Ischemic Preconditioning; LD- Low Dose (0.5 and 1.5 mg/kg;*i.p.*); HD- High Dose (1 and 3 mg/kg;*i.p.*)) Values are expressed as mean ± S.D. (n = 6). Basal denotes RPP measured before global ischemia. 5AR, 30AR, 60AR, and 120AR denote RPP measured 5, 30, 60, and 120 minutes after reperfusion following sustained global ischemia. * = *P* < 0.05 vs Basal;a = *P* < 0.05 vs Normal Control; b = *P* < 0.05 vs Vehicle Control; c = *P* < 0.05 vs IRI Control;d = *P* < 0.05 vs RIPC Sham Control; e = *P* < 0.05 vs RIPC Control. Bold numbers are statistically significant

**Table 5:** Effects of Various Interventions on + dp/dt_max_ and -dp/dt_min_ (mmHg/sec/sec)

**Groups**	**+dp/dt**_max_** (mmHg/sec/sec)**		**-dp/dt**_min_** (mmHg/sec/sec)**	
	**Basal**	**120AR**	**Basal**	**120AR**
Normal Control	6475.93 ± 202.42	6368.45 ± 115.41	-6357.62 ± 202.69	-6341.34 ± 213.68
Vehicle Control	6597.06 ± 300.12	6327.16**±**194.83	-6454.47 ± 277.91	-6478.48**±**131.50
RIPC Sham Control	6499.76 ± 245.59	**2142.97±59.04** ^ab*^	-6286.76 ± 194.92	**-1994.94±93.16** ^ab*^
IRI Control	6387.69 ± 109.10	**2003.26±50.56** ^ab*^	-6228.37 ± 115.60	**-1900.19±107.97** ^ab*^
RIPC Control	6448.55 ± 217.11	**4694.03±117.41** ^cd*^	-6349.93 ± 160.16	**-4405.53±135.87** ^cd*^
AM-630 (LD) + RIPC	6385.70 ± 224.75	**2982.99±158.47** ^abe*^	-6337.35 ± 286.29	**-2688.04±86.07** ^abe*^
AM-630 (HD) + RIPC	6415.19 ± 254.82	**2840.27±152.15** ^abe*^	-6387.48 ± 198.84	**-2605.94±180.54** ^abe*^
BML-275 (LD) + RIPC	6378.45 ± 278.63	**2869.29±108.56** ^abe*^	-6254.47 ± 120.06	**-2777.83±94.52** ^abe*^
BML-275 (HD) + RIPC	6306.92 ± 294.84	**2761.38±83.98** ^abe*^	-6443.59 ± 309.90	**-2680.26±160.82** ^abe*^
AM-630 (HD) *per se*	6441.46 ± 324.69	6324.19**±**126.06^cd^	-6387.12 ± 147.50	-6377.78**±**78.50^cd^
BML-275 (HD) *per se*	6358.75 ± 258.46	6414.12**±**238.41^cd^	-6435.09 ± 326.11	-6332.93**±**97.62^cd^

(Abbreviations: AR – After Reperfusion; IRI- Ischemia-Reperfusion Injury; RIPC- Remote Ischemic Preconditioning; LD- Low Dose (0.5 and 1.5 mg/kg;*i.p.*); HD- High Dose (1 and 3 mg/kg;*i.p.*)) Values are expressed as mean ± S.D. (n = 6). Basal denotes + dp/dt_max_ and -dp/dt_min_ measured before global ischemia. 120AR denotes + dp/dt_max_ and -dp/dt_min_ measured 120 minutes after reperfusion following sustained global ischemia. * = *P* < 0.05 vs Basal;a = *P* < 0.05 vs Normal Control; b = *P* < 0.05 vs Vehicle Control; c = *P* < 0.05 vs IRI Control;d = *P* < 0.05 vs RIPC Sham Control; e = *P* < 0.05 vs RIPC Control. Bold numbers are statistically significant

###  Effects of various interventions on infarct size

**Figure 1:**
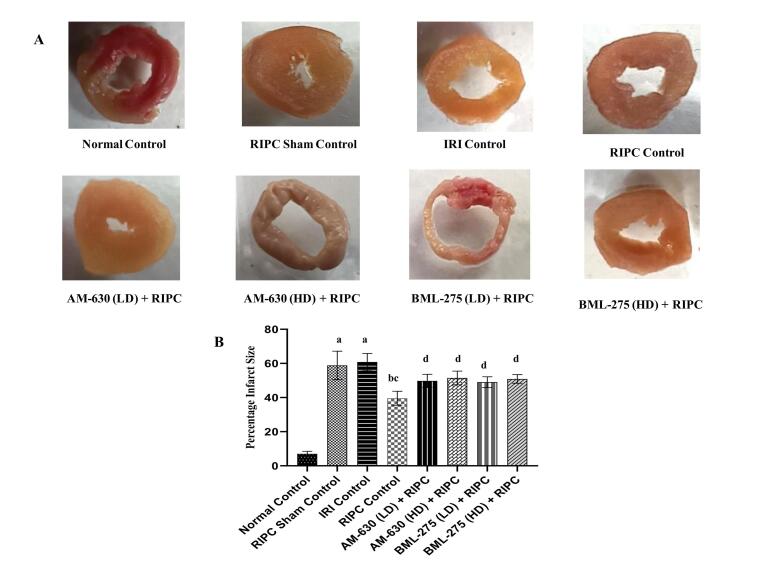
**A)** Photomicrographic presentation of myocardial infarct size using triphenyl tetrazolium chloride (TTC) staining technique. The yellow/white-colored and red-colored region indicates the infarcted and viable areas respectively, in the myocardium of the rat hearts **B).** Effects of various interventions on infarct size measured by area method(Abbreviations: IRI- Ischemia-Reperfusion Injury; RIPC- Remote Ischemic Preconditioning; LD- Low Dose (0.5 and 1.5 mg/kg;*i.p.*); HD- High Dose (1 and 3 mg/kg;*i.p.*)) Values are expressed as mean ± S.D. (n = 6). a = *P < 0.05* vs Normal Control; b = *P < 0.05* vs IRI Control; c = *P < 0.05* vs RIPC Sham Control; d = *P < 0.05* vs RIPC Control

 A significant increase in infarct size was observed in the IRI control group as compared to the normal control group, as assessed by the area method. In contrast, animals subjected to RIPC, exhibited a marked reduction in infarct size relative to the IRI control group. No significant differences were observed between the RIPC sham control group and the IRI control group. However, the cardioprotective effect of RIPC on infarct size was significantly abolished upon pre-treatment with AM-630 (a selective CB_2_R antagonist; 0.5 and 1 mg/kg, *i.p.*) and BML-275 (an AMPK-mediated autophagy inhibitor; 1.5 and 3 mg/kg, *i.p.*) (Figure 1A and 1B).

###  Effects of various interventions on specific biochemical markers of cardiac injury

####  Effects on LDH-1 and CK-MB enzymatic activity in coronary effluent

**Figure 2:**
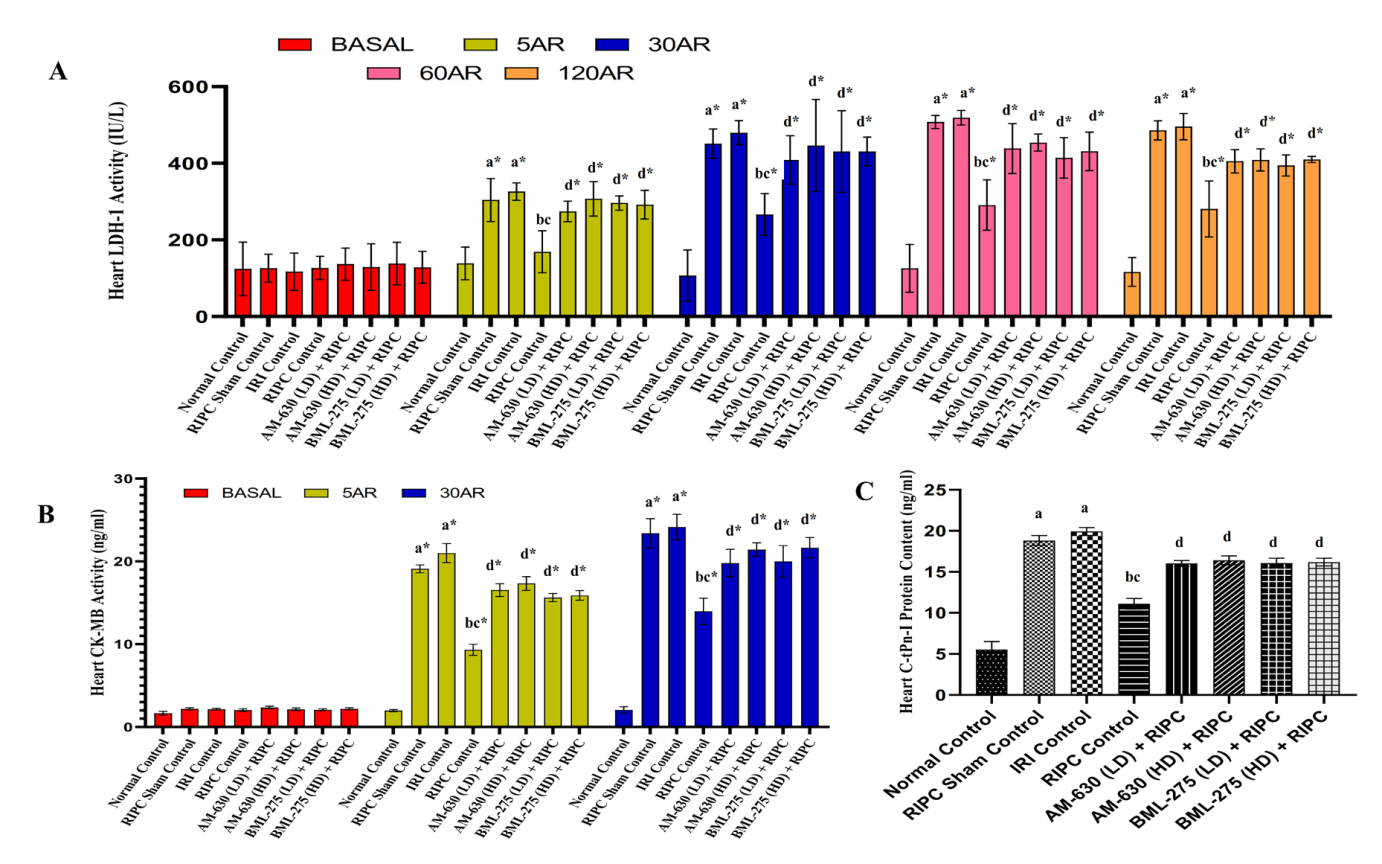
**A, B,C) **Effects of various interventions on LDH-1 and CK-MB activity in coronary effluent collected at different time intervals and C-tPn-I protein content in the heart homogenate (Abbreviations: AR: After reperfusion; CK-MB: Creatine KInase-MB; C-tPn-I: Cardiac troponin-I; IRI- Ischemia-Reperfusion Injury; LDH-I: Lactate dehydrogenase-I; RIPC- Remote Ischemic Preconditioning; LD- Low Dose (0.5 and 1.5 mg/kg;*i.p.*); HD- High Dose (1 and 3 mg/kg;*i.p.*)) Values are expressed as mean ± S.D. (n = 6). LDH-1 and CK-MB activities were measured as IU/L (International Units per Litre) in the coronary effluent collected at different time intervals and C-tPn-I protein content was measured in heart homogenate. Basal denotes LDH-1 and CK-MB measured before global ischemia. 5AR, 30AR, 60AR, and 120AR denote LDH-1 and CK-MB measured 5, 30, 60, and 120 minutes and 5, 30 minutes respectively after reperfusion following sustained global ischemia of 30 min. * = *P < 0.05* vs Basal; a = *P < 0.05* vs Normal Control; b = *P < 0.05* vs IRI Control; c = *P < 0.05* vs RIPC Sham Control; d = *P < 0.05* vs RIPC Control

 A 30 min. episode of global ischemia followed by 120 min. of reperfusion led to a significant elevation in LDH-1 and CK-MB enzyme levels in the coronary effluent collected at specific time frames. For LDH-1, these increased activity were noted at basal (stabilization period), and at 5, 30, 60, and 120 min. after reperfusion; for CK-MB, elevations were observed at basal, 5, and 30 min. post-reperfusion, in comparison to both their respective basal values and those of the normal control groups. Animals subjected to RIPC demonstrated a substantial reduction in both LDH-1 and CK-MB activities compared to the IRI controls, indicating cardioprotection. In contrast, the RIPC sham group failed to show any meaningful decline in these enzymatic markers relative to the IRI group. Notably, pre-treatment with AM-630 (0.5 and 1 mg/kg; *i.p.*) and BML-275 (1.5 and 3 mg/kg; *i.p.*) markedly diminished the beneficial effects of RIPC, as evidenced by significantly restored LDH-1 and CK-MB levels, approaching those observed in the untreated IRI group (Figure 2A and 2B).

####  Effects on C-tPn-I protein content in the heart homogenate

 A 30 min. ischemic insult followed by 120 min. of reperfusion led to a significant elevation in C-tPn-I levels in the heart homogenates of the IRI control group, when compared to the normal animals. In contrast, animals underwent RIPC exhibited a significant attenuation in C-tPn-I content, highlighting the cardioprotective potential of the intervention. The RIPC sham group did not show any meaningful change in C-tPn-I levels relative to the IRI group, indicating the specificity of the protective mechanism. Notably, administration of AM-630 (0.5 and 1 mg/kg; *i.p.*) and BML-275 (1.5 and 3 mg/kg; *i.p.*) prior to RIPC significantly abolished its cardioprotective impact, as reflected by the restoration of C-tPn-I levels towards those observed in the IRI controls (Figure 2C).

###  Effects of various interventions on target-specific oxidative stress parameters in heart homogenate

####  Effects on GSH, catalase and malondialdehyde levels in heart homogenate

**Figure 3:**
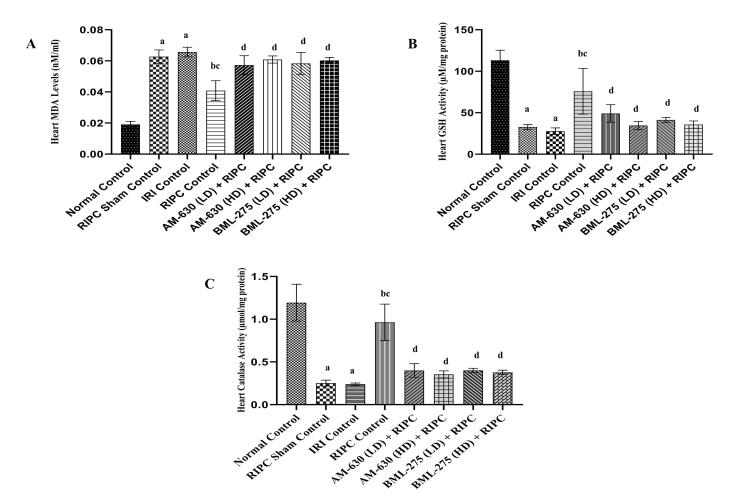
**A, B, C) **Effects of various interventions on MDA levels and GSH and catalase activities in the heart homogenate (Abbreviations: GSH: Glutathione Reductase; IRI- Ischemia-Reperfusion Injury; MDA: Malonadialdehyde; RIPC- Remote Ischemic Preconditioning; LD- Low Dose (0.5 and 1.5 mg/kg;*i.p.*); HD- High Dose (1 and 3 mg/kg;*i.p.*)) Values are expressed as mean ± S.D. (n = 6). a = *P < 0.05* vs Normal Control; b = *P < 0.05* vs IRI Control; c = *P < 0.05* vs RIPC Sham Control; d = *P < 0.05 *vs RIPC Control

 IRI control group animals showed a substantial increase in MDA (Figure 3A) levels and decrease in GSH (Figure 3B) and catalase (Figure 3C) activities in heart homogenate as compared to the normal control group. RIPC witnessed a significant reversal in all aforementioned oxidative stress parameters as compared to the IRI control group animals, reflecting the cardioprotective potential of RIPC. On contrary, RIPC sham group animals did not show any significant improvement in terms of oxidative stress parameters. However, pre-treatment with AM-630 (0.5 and 1 mg/kg; *i.p.*) and BML-275 (1.5 and 3 mg/kg; *i.p.*) significantly abrogated the beneficial effects of RIPC on oxidative stress markers (Figure 3A-3C).

###  Effects of various interventions on inflammatory and fibrotic markers

####  Effects on TNF-α levels in the heart homogenate

**Figure 4:**
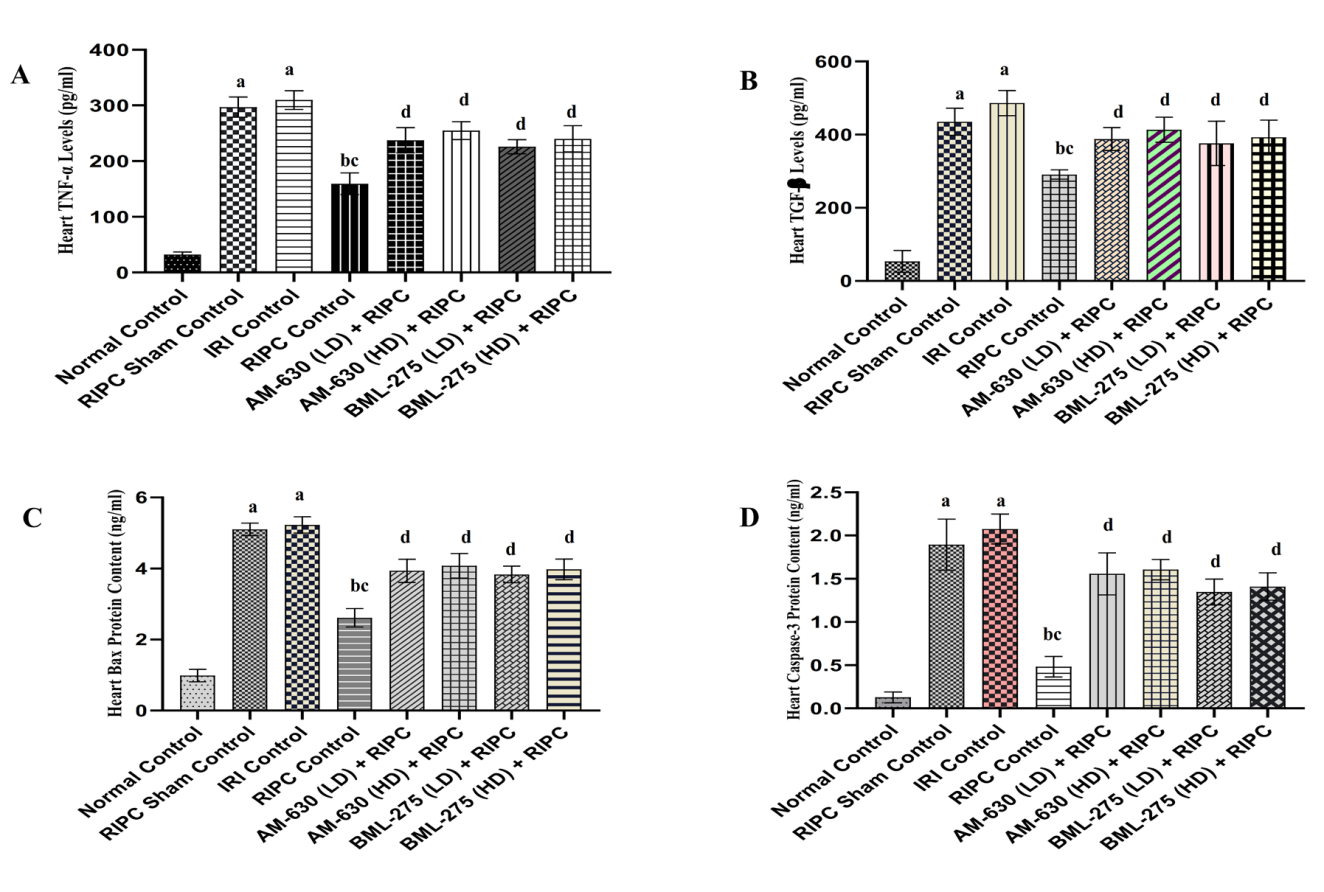
**A, B, C,D). **Effects of various interventions on TNF-α and TGF-β levels and Bax and caspase-3 protein content in the heart homogenate (Abbreviations: IRI- Ischemia-Reperfusion Injury; RIPC- Remote Ischemic Preconditioning; LD- Low Dose (0.5 and 1.5 mg/kg;*i.p.*); HD- High Dose (1 and 3 mg/kg;*i.p.*); TGF-β: Tumor Growth Factor-β; TNF-α: Tumor Necrosis Factor-α) Values are expressed as mean ± S.D. (n = 6). a = *P < 0.05* vs Normal Control; b = *P < 0.05* vs IRI Control; c = *P < 0.05* vs RIPC Sham Control; d = *P < 0.05* vs RIPC Control

 The induction of global ischemia for 30 min., followed by 120 min. of reperfusion, led to a significant surge in TNF-α concentration within the cardiac tissue of IRI control animals, relative to those in the normal group. This inflammatory response was notably mitigated in animals subjected to RIPC, as evidenced by a significant decline in TNF-α levels. However, this suppressive effect on inflammation was absent in the RIPC sham group, where TNF-α levels remained same as observed in the IRI controls. Interestingly, when animals received pre-treatment with AM-630 (0.5 and 1 mg/kg; *i.p.*) and BML-275 (1.5 and 3 mg/kg; *i.p.*), the RIPC mediated attenuation of TNF-α was effectively nullified, underscoring the role of CB_2_R signaling and AMPK regulated autophagy in the observed cardioprotection (Figure 4A).

####  Effects on TGF-β levels in the heart homogenate

 In comparison to the normal group, animals subjected to IRI exhibited a pronounced elevation in TGF-β levels within the cardiac tissue. This pathological increase was significantly attenuated in the RIPC control group animals, suggesting the cardioprotective potential of RIPC. However, animals of the RIPC sham group failed to show any meaningful reduction in TGF-β concentrations when compared with IRI controls. Furthermore, the beneficial effects of RIPC was remarkably abolished after prior administration of AM-630 (0.5 and 1 mg/kg; *i.p.*) and BML-275 (1.5 and 3 mg/kg; *i.p.*), supporting the involvement of CB_2_R and AMPK autophagy in mediating cardioprotective effect of RIPC (Figure 4B).

###  Effects of various interventions on apoptotic markers

####  Effects on Bax and Caspase-3 protein content in the heart homogenate

 A global ischemic insult of 30 min. followed by 120 min. of reperfusion resulted in a pronounced elevation of apoptotic proteins Bax and caspase-3 in the heart homogenates of IRI control group animals when compared to normal control groups. RIPC markedly suppressed the levels of these apoptotic markers in the RIPC control group. In contrast, the RIPC sham group failed to exhibit any significant reduction in Bax and caspase-3 protein levels as compared to the IRI control group. Notably, pre-treatment with AM-630 (0.5 and 1 mg/kg, *i.p.*) and BML-275 (1.5 and 3 mg/kg, *i.p.*) effectively nullified the protective effect of RIPC in terms of significant rise in Bax and caspase-3 levels as compared to RIPC control animals (Figure 4C and 4D).

## Discussion

 This study provides novel insights by being the first to investigate the combined mechanistic involvement of CB_2_R and AMPK mediated autophagy signaling pathways in RIPC-mediated cardioprotection against MIRI. In this study, MIRI was induced using the Langendorff isolated heart model, involving 30 min. of global ischemia followed by 120 minutes of reperfusion with KH buffer.^57^ This *ex-vivo* model reliably measures HR, LVDP, and coronary flow.^58^ Presently, myocardial injury was assessed through infarct size, cardiac biomarkers (LDH-1, CK-MB, C-tPn-I), hemodynamic parameters, oxidative stress markers (MDA, GSH, catalase), and indicators of inflammation and fibrosis (TNF-α, TGF-β respectively) and apoptosis (Bax, caspase-3).

 Thirty minutes of ischemia followed by 120 minutes of reperfusion caused significant myocardial injury, evident from increased TTC-stained infarct size and elevated LDH-1 and CK-MB activities in coronary effluent. Elevated levels of C-tPn-I,, TNF-α, TGF-β, Bax, caspase-3, and MDA, along with reduced GSH and catalase in heart tissue, further confirmed injury and are consistent with our earlier findings^8,59^ and other established literature.^60-62^ RIPC, using four cycles of brief limb ischemia and reperfusion, significantly attenuated MIRI by reducing infarct size, cardiac injury markers, oxidative stress, inflammation, and apoptosis markers, while improving antioxidant enzyme activity and restoring key hemodynamic parameters including HR, LVDP, CFR, RPP, + dp/dt_max_, and -dp/dt_min_.

 The observed improvement in HR and LVDP following RIPC intervention holds important clinical significance. A significant decline in HR and compromised LVDP are manifestations of impaired myocardial function during IRI, reflecting diminished contractile performance. Restoration of HR within physiological limits suggests improved myocardial perfusion and reduced arrhythmic susceptibility. Similarly, the preservation of LVDP indicates better systolic function and myocardial contractility, both of which are essential for maintaining cardiac output during reperfusion. These improvements suggest that RIPC not only limits cellular damage but also helps preserve overall mechanical function of the heart, supporting its potential as a non-invasive, clinically relevant strategy for myocardial protection during cardiac surgeries or acute coronary syndromes.

 Several preclinical ^19,63,64^ and clinical studies ^65,66^ have shown that RIPC effectively reduces infarct size, improves ventricular function, and limits edema and arrhythmias^67^ following MIRI. In our study, RIPC-induced cardioprotection was abolished by AM-630, a selective CB_2_R antagonist, indicating CB_2_R involvement. The endocannabinoid system, comprising CB_1_R, CB_2_R^68^, and their ligands^69^, has emerged as a promising therapeutic target in conditions involving inflammation and tissue injury.^70^ Endocannabinoids have shown protective roles in MIRI ^71^, with CB_2_R involvement confirmed in various IRI models^72^, atherosclerosis.^73^ RIPC-induced cardioprotection is linked to CB_2_R signaling, as demonstrated by studies where CB_2_R antagonists reversed the protective effects of preconditioning stimuli like lipopolysaccharide and heat stress in isolated rat hearts.^74,75^ In our study, the reversal of RIPC-induced cardioprotection by CB_2_R antagonist (AM-630; 0.5 and 1 mg/kg;*i.p.*) suggests a key role of CB_2_R, though its exact cardioprotective mechanisms remain debated and require further investigation.

 In the present study, BML-275 (an AMPK-mediated autophagy inhibitor) nullified RIPC-induced cardioprotection, suggesting a vital role of autophagy. Autophagy, a regulated process of degrading damaged cellular components^31^, is triggered under pathological conditions like nutritional deficiency, ischemia and hypoxia.^32^ Evidence supports its role in attenuating IRI ^33,34,76^, with cardioprotective stimuli such as caloric restriction^77^, exercise^78^, and lipopolysaccharide^79^; activating autophagy via pathways including protein kinase-C (PKC), reactive oxygen species (ROS), NO, and AMPK. Additionally, both ischemic and pharmacological preconditioning has been shown to enhance autophagy, contributing to myocardial protection.^80-82^

 AMPK, a central regulator of energy balance^37^, activates autophagy under stress conditions like IRI.^38-40^ It promotes cell survival by reducing oxidative stress, preserving mitochondria, and maintaining endothelial function during RIPC. In our study, BML-275 (a selective AMPK mediated autophagy inhibitor; 1.5 and 3 mg/kg;*i.p.*), significantly diminished the cardioprotective effects of RIPC in MIRI, indicating that RIPC-mediated cardioprotection is closely linked to AMPK-induced autophagy activation.

 On the basis of aforementioned findings and data in hand, it may be concluded that the current study not only confirm the individual roles of CB_2_R and AMPK-autophagy but also uncover their potential interactive effects, offering a broader mechanistic framework for optimizing RIPC as a therapeutic strategy.

 In addition to the AMPK-autophagy axis, multiple signaling cascades are intricately involved in mediating cardioprotection during IRI. These include the PI3K/Akt, ERK1/2, JAK/STAT, and PKC pathways, all of which intersect with autophagic and apoptotic processes. The interplay between AMPK and these pathways may offer additive or synergistic cardioprotective benefits. For instance, AMPK activation has been shown to cross-talk with Akt signaling to promote mitochondrial integrity and inhibit apoptosis.^83,84^ The current study’s findings on AMPK-mediated autophagy suggest one central mechanism, yet the potential involvement of these additional networks cannot be overlooked and should be explored in future investigations to better understand the broader signaling framework underpinning RIPC-induced cardioprotection. This study utilized the *ex-vivo* Langendorff model (gold standard method to induce MIRI), allowing controlled induction of MIRI but lacking systemic neuro-humoral interactions. We focused on acute post-reperfusion outcomes without assessing long-term cardiac function. While AMPK-mediated autophagy was pharmacologically inhibited using BML-275, specific autophagy markers were not evaluated. These limitations further demands for future *in-vivo* studies with molecular validations and extended time-frame evaluations to better understand RIPC’s cardioprotective mechanisms.

 Although our findings highlight the involvement of CB_2_R activation and AMPK-mediated autophagy in RIPC-induced cardioprotection, the molecular interplay between these pathways remains unclear. CB_2_R may preserve mitochondrial integrity and prevent mitochondrial permeability transition pore (mPTP) opening, further reduces cardiomyocyte apoptosis^41^, while AMPK activation supports mitochondrial biogenesis and autophagy under ischemic stress.^85-87^; their convergence likely regulates redox balance by limiting ROS and enhancing antioxidant defenses. Future studies should investigate how mitochondrial dynamics, redox-sensitive signaling, and transcriptional regulators coordinate these protective effects during RIPC.

## Conclusion

 The current findings support that CB_2_R activation and AMPK-mediated signaling are integral to RIPC-induced cardioprotection. CB_2_R activation offers anti-inflammatory and anti-apoptotic benefits without central side effects, while AMPK enhances myocardial energy balance, autophagy, and mitochondrial function under ischemic stress. Selective modulation of these targets can optimize therapeutic outcomes by minimizing systemic risks. Their combination within the RIPC framework holds translational potential, particularly in acute cardiac care, where non-invasive, organ-protective strategies are critical. Further exploration of these pathways may enable safer, target-specific interventions to reduce IRI in clinical settings.

## Competing Interests

 Authors declare that there is no conflict of interest.

## Ethical Approval

 Experimental protocol was duly approved by Institutional Animal Ethics Committee approval No: 107/Go/ReBi/S/99/CCSEA/2021-10.
